# Barriers and Facilitators for the Use of Patient Lifts by Healthcare Workers: A Scoping Review

**DOI:** 10.3390/ijerph21121659

**Published:** 2024-12-12

**Authors:** Ghassan M. Khairallah, Hani Mowafi, Samar Al-Hajj, Alexandria Brackett, Carine J. Sakr

**Affiliations:** 1 Employee Health Unit, Department of Family Medicine, Faculty of Medicine, American University of Beirut Medical Center, Beirut, Beirut P.O. Box 11-0236, Lebanon; gk44@aub.edu.lb; 2 Department of Emergency Medicine, Faculty of Medicine, Yale University, Yale-New Haven Hospital, New Haven, CT 06501, USA; hani.mowafi@yale.edu; 3 Department of Epidemiology and Population Health, Faculty of Health Sciences, American University of Beirut, Beirut, Beirut P.O. Box 11-0236, Lebanon; sh137@aub.edu.lb; 4 Harvey Cushing/John Hay Whitney Medical Library, New Haven, CT 06501, USA; alexandria.brackett@yale.edu

**Keywords:** patient lifts, safe patient handling and motility, healthcare workers, assistive devices, implementation, ergonomics, occupational hazards, healthcare, health and safety, theory of planned behavior

## Abstract

(1) Background: Patient lifts are evidence-based engineering controls used in Safe Patient Handling Programs to assist healthcare workers in moving patients. They have been shown to be beneficial for both healthcare workers and patients. However, these devices are not consistently used. This review aims to determine the scope of the literature and examine the barriers and facilitators for the use of patient lifts by healthcare workers, on a global level. (2) Methods: Electronic databases, including MEDLINE (Ovid), Embase (Ovid), Global Health (Ovid), CINAHL, Scopus, Web of Science—Core Collection, Cochrane CENTRAL, Trials Register of Promoting Health Interventions, PAIS Index (Proquest), and the gray literature were reviewed. Duplicates were removed, titles and abstracts were screened, full texts were assessed, and the quality of the studies were checked. The analysis was carried out qualitatively using thematic analysis. (3) Results: A total of 57 articles were included in this review. Most studies (71.9%) originated in the US alone, and none originated in low- and middle-income countries. The majority were quantitative studies and were conducted in acute care hospitals. The main identified barriers were equipment-related (e.g., time constraints, device unavailability, and inconvenient storage), followed by cultural and behavioral factors (peer pressure, resistance to change, and occupational socialization), followed by organizational factors (staff shortage and workload). The main identified facilitators were mostly organizational factors (leadership support, minimal lift policy, standardized protocols), followed by cultural and behavioral factors (safety culture and worker’s empowerment), then equipment-related factors (device availability and accessibility). Patient- and worker-related factors were the least mentioned. (4) Conclusion: There is a complex interplay of organizational, equipment-related, and cultural factors shaping the use of lifts by healthcare workers. A multifaceted approach that focuses on enhancing organizational support, fostering a robust safety culture, and ensuring equipment availability is warranted.

## 1. Introduction

Hospitals constitute one of the most hazardous working environments [1]. In addition to being exposed to numerous hazards, such as blood-borne pathogens, communicable diseases, chemicals and cytotoxic drugs, and radioactive material, the nursing staff suffer from ergonomic hazards due to patient handling and repetitive tasks [1,2]. Nurses experience a higher-than-average incidence of work-related musculoskeletal diseases (MSDs): back pain, neck pain, and sprains and other injuries to their upper and lower extremities [2]. According to the US Bureau of Labor Statistics (BLS), the incidence rate of MSDs among registered nurses (RNs) is 46 per 10,000 full-time workers, surpassing the average of all occupations (less than 30 per 10,000 full-time workers) [2]. Nursing assistants record much higher MSD rates (above 150 per 10,000 full-time workers) compared to other healthcare workers [3]. The BLS reported that nearly half (52%) of all non-fatal injuries and illnesses among nurses result from overexertion and bodily reactions (strains, sprains, and MSDs) [2]. Similarly, a study conducted in Lebanon to assess the prevalence and predictors of low back pain among healthcare workers revealed that 54% of nurses suffer from low back pain, and 72% of these cases are attributable to lifting force [4].

Therefore, work-related MSDs constitute a major safety concern at hospitals and healthcare centers. The Occupational Safety and Health Administration (OSHA) recommends that manual lifting of patients be minimized in all cases and eliminated when feasible [5]. Ample evidence suggests that the implementation of a Safe Patient Handling and Motility program (SPHM) reduces injury and absenteeism rates. A SPHM involves a structured framework that incorporates the following evidence-based tactics: engineering controls (patient lift equipment), administrative controls (policies, algorithms, and education), and behavioral controls (unit-based peer coaches) [6]. A patient lift is a mechanical device specially designed to lift and transfer patients who need assistance with their mobility. It typically uses a sling and consists of a frame which is mobile (floor-based) or ceiling-mounted. An electric or hydraulic system powers the lifting mechanism.

Studies have shown that the introduction of a SPHM that incorporates assistive devices at the Veterans Health Administration in the USA resulted in a 30% reduction in injury rates [7]. In addition, the cost–benefit analysis showed a net savings of USD 200,000 per year, and the initial capital investment was recovered in approximately four years [7,8]. The National Institute of Occupational Safety and Health (NIOSH) conducted several studies with multifaceted interventions involving the use of mechanical lifts, safe lifting policy and staff training. The number of injuries from patient transfers decreased by 62%, lost workdays decreased by 86%, restricted workdays decreased by 64%, and workers’ compensation costs decreased by 84% [9].

However, in most hospitals and chronic care facilities, this equipment’s utilization remains suboptimal despite the proven health and economic benefits. Several studies confirmed that nursing staff use the lifts inconsistently, despite their wide availability, indicating a utilization rate of 21% during patient transfer activities [10]. Rates differed by the type of lift/transfer performed [10]. Lift equipment was not used for 82% of patient handling injuries, and higher injury rates were recorded for non-use of equipment versus use (IRR = 4.7) [10,11].

Given the magnitude of the burden of MSDs among nurses related to lifting and manual handling of patients, and the low use of assistive devices, a largely unexploited opportunity exists with regard to understanding the barriers and facilitators for the use of the evidence-based SPHM, specifically patient lifts. Barriers and facilitators can span a range of organizational, cultural and logistical factors. If specific factors influencing healthcare workers’ behavior are identified, and the necessary changes are implemented, healthcare workers may be more likely to use assistive devices and prevent occupational injuries [12,13]. The findings of this study are interpreted through the lens of the Theory of Planned Behavior (TPB), which was proposed by Ajzen in 1991 [14]. According to the TPB, an individual’s behavior is determined by their intention to perform that behavior. The theory identified three key elements that shape an individual’s intention, and hence their behavior: attitudes (positive or negative evaluation of the behavior), subjective norms (perceived social pressures to perform or not to perform the behavior), and perceived behavioral control (the perceived ease or difficulty of performing the behavior) [14].

This review aims to determine the scope of the literature and systematically examine the barriers and facilitators for the use of patient lifts by healthcare workers. Our study seeks to evaluate and synthesize the findings, making them available to facilitate the implementation of relevant interventions that enhance patient and staff safety. Evidence from this review will improve our understanding and guide the implementation of evidence-based approaches to support the use of assistive devices and overcome potential challenges.

## 2. Materials and Methods

This review examined the literature on the barriers and facilitators for the use of assistive devices that were identified in SPHM interventions involving the use of patient lifts, on a global level. A structured methodological approach was adopted as described by the Preferred Reporting Items for Systematic Reviews and Meta-Analyses extension for Scoping Reviews (PRISMA-ScR) guidelines. The study protocol was registered and published in Open Science Framework OSF, registration DOI: https://doi.org/10.17605/OSF.IO/43ZPC (accessed on 1 November 2024).

### 2.1. Scoping Review Framework

This scoping review was guided by the JBI framework, as detailed in the “JBI Manual for Evidence Synthesis” (https://jbi-global-wiki.refined.site/space/MANUAL/355862599/10.1.3+The+scoping+review+framework, accessed on 1 November 2024). The JBI approach to the conduct of scoping reviews (Peters et al., 2015) follows a clear and rigorous process that is suitable for studying our research question. The steps are as follows:

1. Defining and aligning the objective/s and question/s.
2. Developing and aligning the inclusion criteria with the objective/s and question/s.
3. Describing the planned approach to evidence searching, selection, data extraction, and presentation of the evidence.
4. Searching for the evidence.
5. Selecting the evidence.
6. Extracting the evidence.
7. Analyzing the evidence.
8. Presentation of the results.
9. Summarizing the evidence in relation to the purpose of the review, making conclusions and noting any implications of the findings.

### 2.2. Search Strategy

In collaboration with a clinical research and education librarian (AB), the research team developed a comprehensive search strategy. The following nine databases were searched: MEDLINE (Ovid), Embase (Ovid), Global Health (Ovid), CINAHL, Scopus, Web of Science—Core Collection, Cochrane CENTRAL, Trials Register of Promoting Health Interventions and PAIS Index (Proquest). This search combined applicable controlled vocabulary and keyword terms related to patient positioning; devices used to assist with positioning; nurses and other medical staff; and facilitators and barriers (File S1).

Unpublished studies and reports were also searched, along with the gray literature, through sources such as theses and dissertations, conference proceedings, government reports, and organizational websites. Relevant systematic literature reviews were included, and their reference lists were recursively searched for additional studies. Google Scholar was used to review the citing references of all included studies and reports. Consultation with experts in the field helped finalize the search strategy and ensure the inclusion of relevant literature. Additionally, relevant occupational health and healthcare workers-related websites were searched for published and unpublished literature. The Medical Subject Headings (MeSHs) terms used to search the databases are presented in File S1.

### 2.3. Eligibility Criteria

We adopted the PIO framework (population; issue; outcome) as a scoping approach to screen and select eligible studies and include them accordingly. The selection criteria were as follows: (1) Population/setting: Studies involving healthcare workers in both hospitals and chronic care facilities. (2) Issue: Studies evaluating any potential barrier(s) or facilitator(s) for the use of assistive device alone or in combination with any other intervention(s). (3) Study Design: Both quantitative and qualitative studies (observational, quasi-experimental, experimental, and mixed-methods studies), systematic reviews and meta-analyses. (4) Language: Studies published in the English language, as well as studies published in other languages with an available translation or abstract written in English. (5) Publication status: Peer-reviewed journal articles, conference proceedings, gray literature, and unpublished studies. (6) Time frame: Studies published at any time. (7) Geographical scope: global.

### 2.4. Data Screening, Selection, and Extraction

Articles revealed by the search were exported to the Covidence software platform [14]. Duplicates were removed and screening of titles and abstracts was performed independently and in duplicate by two authors (GK and CS) to select potentially eligible studies [15]. Following the identification of eligible articles, two authors (GK and CS) independently evaluated the full text of relevant articles and abstracted data. Disagreements were resolved by consultation with a third reviewer (HM) in order to reach consensus [16]. Abstracted data include study author(s), year of publication, journal, country, language, format, design, setting, population, inclusion and exclusion criteria, sample size, aim(s), outcome(s), barriers and/or facilitators, intervention(s), and main findings. An Excel spreadsheet was used to create a data charting table (File S2). Each row represented a study, and each column represented specific extracted information [17]. This method allowed for easy organization and sorting of extracted data.

### 2.5. Data Analysis and Synthesis

All results underwent double data entry by two authors (GK and CS) to ensure accuracy. Barriers and facilitators for the use of assistive devices by healthcare workers were examined using the PIO framework. The data were categorized into meaningful themes by identifying recurring concepts and patterns across the included studies using an Excel spreadsheet [17]. The studies were assessed for methodologic quality using the JBI checklist (https://jbi.global/critical-appraisal-tools, accessed on 1 November 2024) and the mixed-methods appraisal tool (MMAT). The quality of each study was assessed independently by two authors (GK and CS), and discrepancies were resolved by discussion and consensus. The results were synthesized through descriptive data analysis (country, study design, sample size, population’s characteristics, barriers and facilitators categories…) and thematic analysis. The literature was then mapped and visually represented in tables and graphs, providing a clear overview of the body of research and study outcomes. A narrative synthesis of the findings was provided [17].

## 3. Results

### 3.1. Study Characteristics

**Figure 1:**
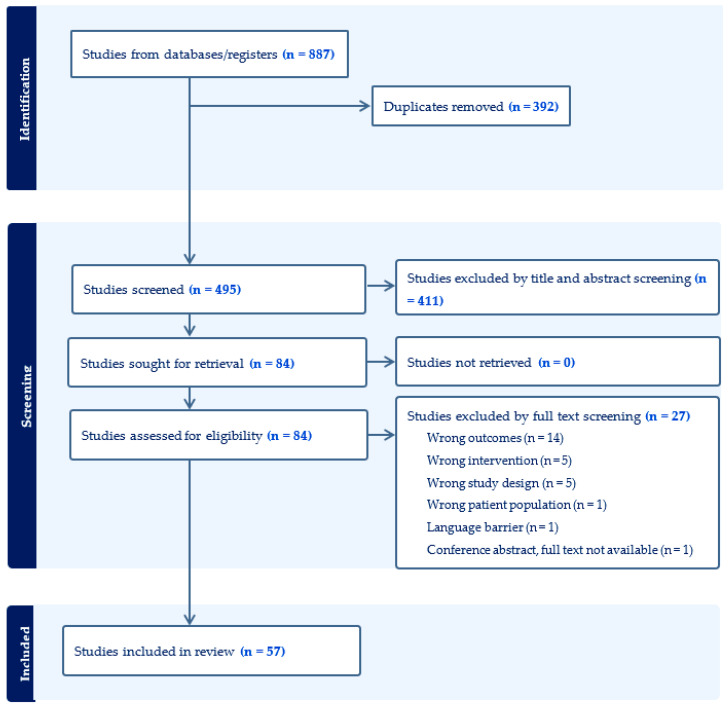
Preferred Reporting Items for Systematic Reviews and Meta-Analyses (PRISMA) flow diagram.

The search yielded a total of 887 articles. After duplicates had been identified and removed, 495 unique articles were included (Figure 1). Title and abstract screening resulted in the exclusion of 411 articles, and the remaining 84 articles were assessed for eligibility based upon the objectives of this scoping review. After that, 27 articles were further excluded by full-text screening, resulting in a final total of 57 articles (Figure 1). The reasons for the full-text exclusion phase were mainly due to wrong outcomes (i.e., the study did not evaluate barriers nor facilitators), or wrong interventions (i.e., the study did not primarily address the lift machine), or the wrong study design (i.e., the study design did not adequately match the research question). All included studies were of medium to high quality (File S3).

In total, 41 of the 57 articles originated in the USA (71.9%), 6 (10.5%) in the UK, 3 (5.3%) each in Denmark and the Netherlands, 2 (3.5%) in Canada, and 1 (1.8%) in both Brazil and South Korea. The oldest study was published in 1992 and the newest was published in 2022. The majority of the studies were quantitative (59.6%), followed by review articles (15.8%), qualitative (14%), mixed methods (7%), and brief reports (3.5%). Most of the studies investigated both barriers and facilitators for the use of assistive devices by healthcare workers (59.6%), followed by facilitators only (21.1%) and barriers only (19.3%). Twenty-eight (49.1%) studies were conducted in an acute care hospital setting, followed by nine (15.8%) that were conducted in chronic care facilities and nursing homes.

### 3.2. Barriers and Facilitators Sub-Analysis

Under each broad category of barriers or facilitators, the retrieved studies were classified based on their overarching themes, including equipment-related factors, patient-related factors, worker-related factors, organizational factors, and behavioral/cultural factors. Under each theme, the codes are displayed in descending order of citation frequency (Table 1 and Table 2). The main barriers for device use were primarily attributed to equipment-related (logistic) factors, followed by behavioral and cultural factors, then organizational factors. The main facilitators for device use were primarily attributed to organizational factors, followed by behavioral and cultural factors, then equipment-related factors.

**Figure 2:**
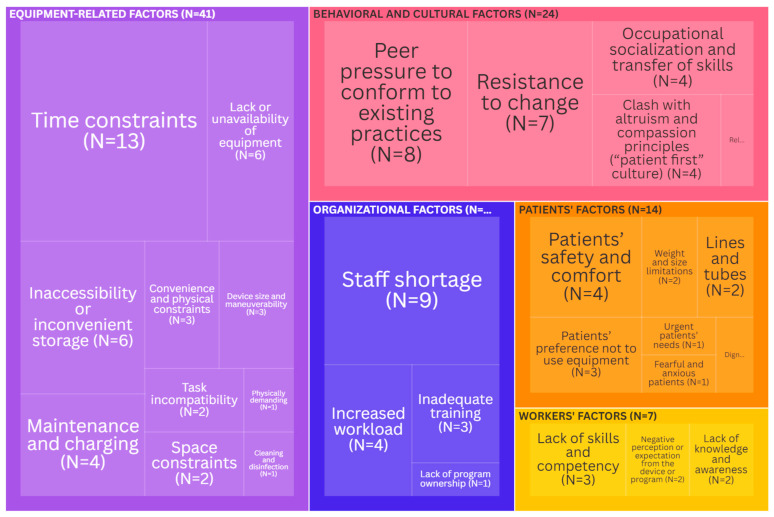
Tree map depicting the key barriers (the size of each rectangle is indicative of the citation frequency in the literature). An interactive version of this treemap is available online: https://public.flourish.studio/visualisation/19954214/ (accessed on 1 November 2024). Through the interactive version, users can futher explore the data. Created with flourish.studio (https://flourish.studio, accessed on 1 November 2024).

Barriers: Equipment-related factors were the most cited (41 times) barriers for the use of the lifts (Figure 2). These factors were mainly related to time constraints [12,18,19,20,21,22,23,24,25,26,27,28,29], followed by the lack or unavailability of equipment [12,21,22,30,31,32] and inaccessibility or inconvenient storage [12,18,20,21,26,32]. Behavioral and cultural factors emerged as an important theme (cited 24 times) preventing the use of lifts. The literature revealed several key aspects of this theme: peer pressure to conform to existing practices [10,21,22,26,33,34,35,36], resistance to change [21,26,27,37,38,39,40], and occupational socialization and transfer of skills [34,36,38,41]. Organizational factors were cited 17 times in the literature as potential barriers. This was mainly due to staff shortages [10,12,18,19,20,21,22,42,43] and increased workload [12,21,42,43]. Patient- and worker-related factors were the least mentioned in the literature. The two most common patient-related barriers were safety and comfort [18,19,23,44] and patients’ preference not to use equipment [20,30,45]. The most common worker-related barriers were a lack of skill and insufficient competency [18,21,24].

**Figure 3:**
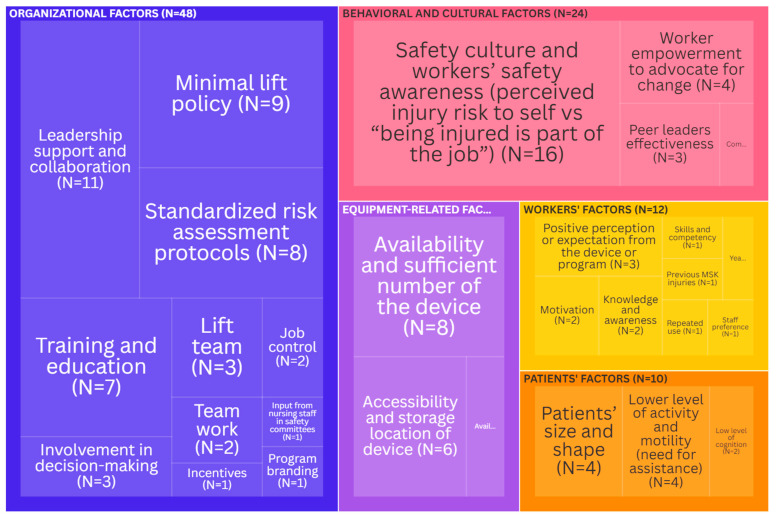
Tree map depicting the key facilitators (the size of each rectangle is indicative of the citation frequency in the literature). An interactive version of this treemap is available online: https://public.flourish.studio/visualisation/19954212/ (accessed on 1 November 2024). Through the interactive version, users can futher explore the data. Created with flourish.studio (https://flourish.studio, accessed on 1 November 2024).

Facilitators: Organizational factors were the most cited (48 times) facilitators for the use of the assistive device (Figure 3). The presence of strong leadership support and collaboration emerged as the top influencing factor [21,24,25,30,38,46,47,48,49,50,51]. It was followed by the presence of a minimal lift policy (legislation) [7,31,44,52,53,54,55,56,57], and of standardized risk assessment protocols and standards [7,20,33,35,46,50,52,58]. The training and education of healthcare workers [38,39,43,47,52,56,59] were also important determinants of lift use. Behavioral and cultural factors emerged as important facilitators (cited 24 times) for lifts use. Safety culture (climate) and workers’ safety awareness, including the perceived risk of self-injury, versus the acceptance of injury as “part of the job” [6,21,24,30,36,41,43,48,53,60,61,62,63,64,65,66] were the most mentioned. Worker empowerment [6,26,38,43] was revealed to be an important determinant as well. Equipment-related factors were less commonly referred to as facilitators (cited 16 times): these included the availability and sufficient number of devices [10,25,28,43,50,52,63,64], the accessibility and storage location of devices [10,25,28,52,64,67], and the availability of equipment supplies [10,47]. Patients’ and workers’ factors were the least mentioned. The two most common facilitators due to patients’ factors were patients’ size and shape [10,28,42,61] and a lower level of activity and motility (assistance need) [10,28,42,61]. The most common worker-related facilitator was a positive perception or expectation of the device or program [65,68,69].

**Table 1:** Data summary table for key barriers.

Factor	Codes	Frequency	References
Organizational	Staff shortage	9	[10,12,18,19,20,21,22,42,43]
	Increased workload	4	[12,21,42,43]
	Inadequate training	3	[20,31,46]
	Lack of program ownership	1	[38]
Equipment-related	Time constraints	13	[12,18,19,20,21,22,23,24,25,26,27,28,29]
	Lack or unavailability of equipment	6	[12,21,22,30,31,32]
	Inaccessibility or inconvenient storage	6	[12,18,20,21,26,32]
	Maintenance and charging	4	[20,28,33,47]
	Convenience and physical constraints	3	[24,25,31]
	Device size and maneuverability	3	[29,32,52]
	Task incompatibility	2	[20,61]
	Space constraints	2	[20,26]
	Physically demanding	1	[18]
	Cleaning and disinfection	1	[20]
Behavioral/cultural	Peer pressure to conform to existing practices	8	[10,21,22,26,33,34,35,36]
	Resistance to change	7	[21,26,27,37,38,39,40]
	Occupational socialization and transfer of skills	4	[34,36,38,41]
	Clash with altruism and compassion principles (“patient first” culture)	4	[19,40,41,60]
	Reluctant to challenge physicians’ requests	1	[39]
Patient-related	Patients’ safety and comfort	4	[18,19,23,44]
	Patients’ preference not to use equipment	3	[20,30,45]
	Weight and size limitations	2	[19,20]
	Lines and tubes	2	[21,61]
	Urgent patients’ needs	1	[12]
	Fearful and anxious patients	1	[61]
	Dignity concerns	1	[62]
Worker-related	Lack of skills and competency	3	[18,21,24]
	Negative perception or expectation from the device or program	2	[32,59]
	Lack of knowledge and awareness	2	[21,24]

**Table 2:** Data summary table for key facilitators.

Factor	Codes	Frequency	References
Organizational	Leadership support and collaboration	11	[21,24,25,30,38,46,47,48,49,50,51]
	Minimal lift policy	9	[7,31,44,52,53,54,55,56,57]
	Standardized risk assessment protocols	8	[7,20,33,35,46,50,52,58]
	Training and education	7	[38,39,43,47,52,56,59]
	Involvement in decision-making (participatory approach)	3	[38,48,70]
	Lift team	3	[43,59,71]
	Job control	2	[38,63]
	Team work	2	[26,51]
	Incentives	1	[38]
	Input from nursing staff in safety committees	1	[43]
	Program branding	1	[38]
Equipment-related	Availability and sufficient number of the device	8	[10,25,28,43,50,52,63,64]
	Accessibility and storage location of device	6	[10,25,28,52,64,67]
	Availability of equipment supplies	2	[10,47]
Behavioral/cultural	Safety culture and workers’ safety awareness (perceived injury risk to self vs. “being injured is part of the job”)	16	[6,21,24,30,36,41,43,48,53,60,61,62,63,64,65,66]
	Worker empowerment to advocate for change	4	[6,26,38,43]
	Peer leaders (coaches) effectiveness	3	[6,51,57]
	Communication and social marketing	1	[37]
Patient-related	Patients’ size and shape	4	[10,28,42,61]
	Lower level of activity and motility (need for assistance)	4	[10,28,42,61]
	Low level of cognition	2	[10,61]
Worker-related	Positive perception or expectation from the device or program	3	[65,68,69]
	Motivation	2	[25,58]
	Knowledge and awareness	2	[50,59]
	Skills and competency	1	[51]
	Previous MSK injuries	1	[58]
	Years of experience	1	[28]
	Repeated use	1	[21]
	Staff preference	1	[10]

## 4. Discussion

To the best of our knowledge, to date, this scoping review is the most comprehensive study assessing the use of safe patient handling devices. It systematically examined the literature related to barriers and facilitators for healthcare workers’ use of patient lifts. It further synthesized the evidence on the various factors impacting device use and the adoption of safe handling practices. This review illustrates how the complex interplay between equipment, workers, patients, organization and culture determines healthcare workers’ use of lifts.

Our findings demonstrate that organizational, equipment-related and behavioral/cultural factors were the most frequently cited themes influencing the use of patient lifts by healthcare workers. These factors, especially the organizational and behavioral/cultural ones, are often closely interrelated. Organizational factors, particularly strong leadership and collaboration, minimal lift policy, standardized risk assessment protocols, and adequate training were pivotal facilitators, while the staff shortage and high workload posed significant barriers. This is consistent with the evidence-based participatory approach that involves workers in decision-making and education to re-design their work and promote health and wellbeing [72]. Equipment-related factors, mainly the availability of the device and the ability to access it in a timely manner, underscore the practical importance of logistical considerations. Behavioral and cultural factors emerged as a theme of paramount importance. In fact, the presence of a safety culture and of injury risk awareness facilitate the use of lifts by healthcare workers, as they are more likely to prioritize their own safety when supported by a positive safety climate. For some healthcare workers, culture is “the way things are done around here” [40]. In the reviewed literature, these workers tend to consider that “injury is just part of the job” and accept it [21]. They think that the use of lifts clashes with the altruism principle and the “patient-first” culture [36,40]. This shows the deep-seated influence of workplace climate (norms) and socialization in shaping healthcare workers’ behavior with regard to the use of the equipment. Peer pressure to conform to existing practices (manual lifting), resistance to change, and the effect of occupational socialization are therefore important barriers. This highlights how social dynamics and group behavior could either support or hinder the adoption of safe patient handling practices. Occupational socialization reflects how the process of learning and integrating new skills related to equipment use was influenced by informal training and the workplace environment [41]. Workers who were not adequately socialized into safe handling practices early in their careers were less likely to adopt lift use later on. In contrast, worker- and patient-related factors, while relevant, were less reported in the literature. Our results concur with those of a systematic review that showed that environmental barriers and facilitators are more important than individual ones during implementation of primary preventive interventions in patient handling [25].

**Figure 4:**
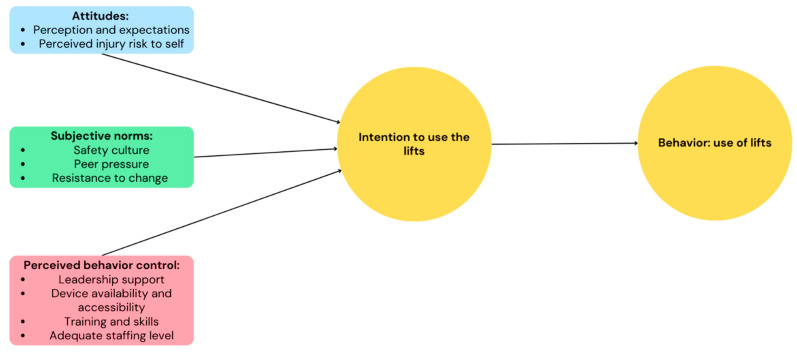
Theory of Planned Behavior applied to healthcare workers’ use of patient lifts.

The TPB’s three key elements that shape individual’s intention and, hence, behavior (attitudes, subjective norms, and perceived behavior control) [14], align with the findings of this study. In the context of this review, healthcare workers’ attitudes towards equipment use are highlighted by the perceived usefulness and expectations of said equipment, and by perceived injury risk, as opposed to the idea that “being injured is part of the job”. Subjective norms include safety culture, peer pressure, and resistance to change. Perceived behavior control is illustrated by a strong leadership support, device availability and accessibility, adequate training and skills, and an adequate staffing level. Therefore, the combination of these three elements influences whether healthcare workers use the lifts (Figure 4).

There is a notable gap in the literature when it comes to studies conducted in low- and middle-income countries (LMICs). This gap in the literature highlights the need for more research in these settings, since the context is different from that of high-income countries. In LMICs, the healthcare system faces significant resource constraints, poor occupational health and safety standards, political instabilities, and lower organizational support, all of which can influence the availability and adoption of safe patient handling practices, including assistive devices. A study conducted to assess occupational health and safety accreditation status among Lebanese hospitals found that 56% of the participating private hospitals were accredited [73], and this number is far below that of the US [74]. The study showed that accredited private hospitals reported better health and safety performance than non-accredited hospitals [73]. In addition, cultural perceptions of manual work, patient care, and the role of healthcare workers may differ in LMICs. Therefore, it is necessary to explore how these unique factors influence safe patient handling practices.

This scoping review has several strengths. It employed a rigorous search strategy on a global level, aiming to map all the existing literature and identify gaps. The review process was conducted independently by two reviewers, and conflicts were solved by reaching a consensus or by a third reviewer. The review assessed the quality of the included studies, ensuring a medium to high level of evidence. It also included the gray literature (reports and academic dissertations) in addition to peer-reviewed articles, allowing it to capture a broader perspective. However, despite the rigorous search strategy, it faced limitations such as the possibility of missing important studies due to language barriers. Furthermore, the absence of published studies from LMICs limits the generalizability of our findings. Also, none of the retrieved articles addressed the problem from the perspective of other stakeholders (managers, policy makers, or patients), potentially overlooking important data. Finally, since the analysis was conducted in qualitative terms, we could not assess how the identified factors interact with each other to obtain a more nuanced understanding of their impact on the use of lifts.

The results of this scoping review have several implications for policy and practice. A multifaceted intervention based on participatory ergonomics, investment in healthcare infrastructure, and promotion of a safety culture is warranted. The following recommendations can be considered when developing an effective safe patient handling and motility program involving engineering controls:

On an organizational level: Hospitals and chronic care facilities need to strengthen organizational support, including management support, for healthcare workers and involve them in the decision-making process. Healthcare institutions should offer effective training and education programs for their workers to enhance their competency and comfort with safe patient handling. Institutions need to ensure that staffing is adequate, since understaffing is a barrier to lift use.

On an operational level: Investing in the healthcare infrastructure is essential to ensure the availability of a sufficient number of lifts under the disposition of healthcare workers. These lifts should also be stored in an accessible location and easily retrieved and manipulated in order to avoid any lost time at work.

On a cultural level: Fostering a culture of safety is essential. Empowering healthcare workers and supporting the use of assistive devices can mitigate the impact of peer pressure and resistance to change.

Finally, future research in resource-constrained settings such as LMICs is needed to develop culturally relevant strategies to promote safe patient handling practices. More qualitative studies are needed to gain an in-depth understanding of the unique economic, social, and cultural factors affecting LMICs that will influence the use of assistive devices. This can facilitate the development of more tailored interventions to meet the specific needs of these countries.

## 5. Conclusions

This scoping review provides a comprehensive understanding of the barriers and facilitators for the use of patient lifts by healthcare workers, on a global level. The included studies revealed the complex interplay of organizational, equipment-related, and cultural factors in shaping the adoption and use of assistive devices in safe patient handling practices. Strong leadership support, effective training and education, and a positive safety culture emerged as key facilitators, while logistical barriers, staffing level, peer pressure to conform to existing practices, and resistance to change hindered usage. Addressing these challenges require a multifaceted approach that focuses on enhancing organizational support, fostering a robust safety culture, and ensuring the availability of a sufficient amount of well-maintained equipment. Future research in resource-constrained settings is warranted.

## Data Availability

Data were compiled though Covidence, https://www.covidence.org (accessed on 1 November 2024) and will be available upon request.

## Supplementary Materials

The following supporting information can be downloaded at https://www.mdpi.com/article/10.3390/ijerph21121659/s1, Please refer to File S1 (search strategy), File S2 (data abstraction), and File S3 (quality appraisal).
