# Supplementary material for: Barriers and Facilitators for the Use of Patient Lifts by Healthcare Workers: A Scoping Review
Source: Int J Environ Res Public Health. 2024 Dec 12;21(12):1659. doi: 10.3390/ijerph21121659 (PMC11675142; doi:10.3390/ijerph21121659)
Supplement: Supplementary file 1 [file ijerph-21-01659-s001.zip › File S1.pdf]

Barriers and Facilitators for the Use of Patient Lifts by Healthcare Workers: A Scoping Review

File S1

Methods description:
Search Strategy

In collaboration with a clinical research and education librarian (AB), the research team developed a comprehensive search of the literature across nine databases MEDLINE (Ovid), Embase (Ovid), Global Health (Ovid), CINAHL, Scopus, Web of Science-Core Collection, Cochrane CENTRAL, Trials Register of Promoting Health Interventions and PAIS Index (Proquest). This search combined applicable controlled vocabulary and keyword terms related to patient positioning, devices to assist in positioning, nurses and other medical staff, and facilitators and barriers.

Table s1: MeSH terms used to search the databases

Search group	Terms
Intervention	"Assistive technology" OR "Assistive technologies" OR "Assistive device*" OR "Assistive aid*" OR "Assistive equipment*" OR "Transfer aid*" OR "Transfer device*" OR "Patient lift*" OR "Lift machine*" OR "Lift device*" OR "Lifting machine*" OR "Lifting device*" OR "Lifter*" OR "Ceiling lift*" OR "Hoyer lift*" OR "Floor lift*" OR "Safe patient handling" OR "SPH" OR "Safe patient handling and mobility" OR "SPHM"
Users	"Nurse*" OR "Registered nurse*" OR "Practical nurse*" OR "Nurse assistant*" OR "Nursing staff" OR "Orderlies" OR "Medical staff" OR "Healthcare worker*" OR "Healthcare professional*" OR "Healthcare personnel" OR "Healthcare provider*" OR "Caregiver*" OR "hospital worker*" OR
Barriers &/or Facilitators	"Barrier*" OR "Misuse" OR "Obstacle*" OR "Perception*" OR "Attitude*" OR "Facilitator*" OR "Acceptance" OR "Predictor*" OR "Use" OR "Usage"

Plurals were allowed by using wildcards (*)

Search Strategies for appendix (use all or MEDLINE)

Ovid MEDLINE(R) ALL <1946 to August 07, 2023>
https://ovidsp.ovid.com/ovidweb.cgi?T=JS&NEWS=N&PAGE=main&SHAREDSEARCHID=51fK21ZQ1THthTLN7PRLJqxnQAFmF2frRq2PpuilR7li3ibO88EF0ZQj0FjBaC5Mj

1 "Moving and Lifting Patients"/ or exp Patient Positioning/ 9261
2 (Patient* adj3 (Moving* or Lifting* or positioning* or Repositioning* or Handling*)).tw,kw. 10662
3 1 or 2 18474
4 (ceiling lift* or floor lifter* or Hoyer lift*).tw,kw. 38
5 ((lift or lifting or assistive or handling) adj2 (device* or instrument* or technolog* or aid or equipment*)).tw,kw. 7879
6 is.fs. 688662
7 Occupational Diseases/pc [Prevention & Control] 17548
8 Self-Help Devices/ 5707
9 Safety Management/mt [Methods] 4573
10 4 or 5 or 6 or 7 or 8 or 9 719832
11 nursing assistants/ or exp physical therapist assistants/ or physician assistants/ or medical staff/ or nurses/ or nursing staff/ or occupational therapists/ or physical therapists/ or exp physicians/ 255084
12 (nurse or nurses).tw,kw. 307534
13 (nursing adj1 (staff or personnel or assistant*)).tw,kw. 18752
14 (physical therapist* or occupational therapist* or physician* or clinician).tw,kw. 542121
15 ((medical or hospital or healthcare) adj1 (staff or personnel or assistant* or professional*)).tw,kw. 87078
16 11 or 12 or 13 or 14 or 15 1017211
17 (Facilitat* or Accept* or Predictor* or "Use" or Usage).tw,kw. 4871336
18 (Barrier* or Misuse or "non-use" or Obstacle* or Perception* or Attitude*).tw,kw. 943252
19 px.fs. 1184274
20 17 or 18 or 19 6310275
21 3 and 10 and 16 and 20 207

Embase <1974 to 2023 August 07>
https://ovidsp.ovid.com/ovidweb.cgi?T=JS&NEWS=N&PAGE=main&SHAREDSEARCHID=63exeDB9V1nA67AdpUJ73O8i54cOCYcarijEFGGDICaSVO5P0TQXf5ZdkqNXEuJ71

1 patient lifting/ or patient positioning/ 23910
2 (Patient* adj3 (Moving* or Lifting* or positioning* or Repositioning* or Handling*)).tw,kw. 16676
3 1 or 2 35542
4 (ceiling lift* or floor lifter* or Hoyer lift*).tw,kw. 60
5 ((lift or lifting or assistive or handling) adj2 (device* or instrument* or technolog* or aid or equipment*)).tw,kw. 10850
6 is.fs. 0
7 occupational disease/pc [Prevention] 10920
8 self help device/ 2963

9 4 or 5 or 6 or 7 or 8 23049
10 paramedical personnel/ or health practitioner/ or exp nurse/ or nursing assistant/ or nursing staff/ or occupational therapist/ or physiotherapist/ or physiotherapist assistant/ or physician assistant/ or exp physician/ 1277851
11 (nurse or nurses).tw,kw. 373123
12 (nursing adj1 (staff or personnel or assistant*)).tw,kw. 27504
13 (physical therapist* or occupational therapist* or physician* or clinician).tw,kw. 774996
14 ((medical or hospital or healthcare) adj1 (staff or personnel or assistant* or professional*)).tw,kw. 122743
15 10 or 11 or 12 or 13 or 14 1969727
16 (Facilitat* or Accept* or Predictor* or "Use" or Usage).tw,kw. 6332043
17 (Barrier* or Misuse or "non-use" or Obstacle* or Perception* or Attitude*).tw,kw. 1156519
18 16 or 17 7112373
19 3 and 9 and 15 and 18 132

Global Health <1910 to 2023 Week 30>
https://ovidsp.ovid.com/ovidweb.cgi?T=JS&NEWS=N&PAGE=main&SHAREDSEARCHID=5bSHm8RwAOrNZvYlTMxmOnZzs9yk0H3igz1GxspX7FxZzTM2wypMrhl6olRZpntqi
1 (Patient* adj3 (Moving* or Lifting* or positioning* or Repositioning* or Handling*)).mp. 565
2 (ceiling lift* or floor lifter* or Hoyer lift*).mp. 1
3 ((lift or lifting or assistive or handling) adj2 (device* or instrument* or technolog* or aid or equipment*)).mp. 675
4 2 or 3 675
5 health care workers/ or nurses/ or physicians/ 85193
6 (nurse or nurses).mp. 33627
7 (nursing adj1 (staff or personnel or assistant*)).mp. 2660
8 (physical therapist* or occupational therapist* or physician* or clinician).mp. 72193
9 ((medical or hospital or healthcare) adj1 (staff or personnel or assistant* or professional*)).mp. 21114
10 5 or 6 or 7 or 8 or 9 146092
11 (Facilitat* or Accept* or Predictor* or "Use" or Usage).mp. 882419
12 (Barrier* or Misuse or "non-use" or Obstacle* or Perception* or Attitude*).mp. 212741
13 11 or 12 1013345
14 1 and 4 and 10 and 13 3

CINAHL, 206 results
https://search.ebscohost.com/login.aspx?direct=true&db=ccm&bquery=(((MH+%26quot%3bPatient+Handling%2b%26quot%3b))+OR+(Patient*+N3+(Moving*+OR+Lifting*+OR+positioning*+OR+Repositioning*+OR+Handling*)))+AND+(((MH+%26quot%3bLifting+and+Transfer+Equipment%2b%26quot%3b))+OR+(ceiling+lift*+OR+floor+lifter*+OR+Hoyer+lift*)+OR+((lift+OR+lifting+OR+assistive+OR+handling)+N2+(device*+OR+instrument*+OR+technolog*+OR+aid+OR+equipment*)))+AND+(((MH+%26quot%3bAllied+Health+Personnel%2b%26quot%3b)+OR+(MH+%26quot%3bExpert+Clinicians%2b%26quot%3b)+OR+(MH+%26quot%3bMedical+Staff%2b%26quot%3b)+OR+(MH+%26quot%3bNurses%2b%26quot%3b)+OR+(M

H+%26quot%3bPhysicians%2b%26quot%3b))+OR+(nurse+OR+nurses)+OR+(nursing+N1+(staff+OR+personnel+OR+assistant*))+OR+(physical+therapist*+OR+occupational+therapist*+OR+physician*+OR+clinician)+OR+((medical+OR+hospital+OR+healthcare)+N1+(staff+OR+personnel+OR+assistant*+OR+professional*)))+AND+((Facilitat*+OR+Accept*+OR+Predictor*+OR+%26quot%3bUse%26quot%3b+OR+Usage)+OR+(Barrier*+OR+Misuse+OR+%26quot%3bnon-use%26quot%3b+OR+Obstacle*+OR+Perception*+OR+Attitude*))&type=1&searchMode=Standard&site=ehost-live

Query Limiters/Expanders Last Run Via Results

S17 S3 AND S7 AND S13 AND S16 Expanders - Apply equivalent subjects
Search modes - Boolean/Phrase Interface - EBSCOhost Research Databases
Search Screen - Advanced Search
Database - CINAHL Complete 206

S16 S14 OR S15 Expanders - Apply equivalent subjects
Search modes - Boolean/Phrase Interface - EBSCOhost Research Databases
Search Screen - Advanced Search
Database - CINAHL Complete 2,164,909

S15 Barrier* or Misuse or "non-use" or Obstacle* or Perception* or Attitude* Expanders - Apply equivalent subjects
Search modes - Boolean/Phrase Interface - EBSCOhost Research Databases
Search Screen - Advanced Search
Database - CINAHL Complete 657,378

S14 Facilitat* or Accept* or Predictor* or "Use" or Usage Expanders - Apply equivalent subjects
Search modes - Boolean/Phrase Interface - EBSCOhost Research Databases
Search Screen - Advanced Search
Database - CINAHL Complete 1,712,865

S13 S8 OR S9 OR S10 OR S11 OR S12 Expanders - Apply equivalent subjects
Search modes - Boolean/Phrase Interface - EBSCOhost Research Databases
Search Screen - Advanced Search
Database - CINAHL Complete 1,170,341

S12 (medical or hospital or healthcare) N1 (staff or personnel or assistant* or professional*) Expanders - Apply equivalent subjects
Search modes - Boolean/Phrase Interface - EBSCOhost Research Databases
Search Screen - Advanced Search
Database - CINAHL Complete 103,903

S11 physical therapist* or occupational therapist* or physician* or clinician Expanders - Apply equivalent subjects
Search modes - Boolean/Phrase Interface - EBSCOhost Research Databases
Search Screen - Advanced Search

Database - CINAHL Complete 449,584

S10 nursing N1 (staff or personnel or assistant*) Expanders - Apply equivalent subjects
Search modes - Boolean/Phrase Interface - EBSCOhost Research Databases
Search Screen - Advanced Search
Database - CINAHL Complete 50,355

S9 nurse or nurses Expanders - Apply equivalent subjects
Search modes - Boolean/Phrase Interface - EBSCOhost Research Databases
Search Screen - Advanced Search
Database - CINAHL Complete 581,184

S8 (MH "Allied Health Personnel+") OR (MH "Expert Clinicians+") OR (MH "Medical Staff+") OR (MH "Nurses+") OR (MH "Physicians+") Expanders - Apply equivalent subjects
Search modes - Boolean/Phrase Interface - EBSCOhost Research Databases
Search Screen - Advanced Search
Database - CINAHL Complete 479,762

S7 S4 OR S5 OR S6 Expanders - Apply equivalent subjects
Search modes - Boolean/Phrase Interface - EBSCOhost Research Databases
Search Screen - Advanced Search
Database - CINAHL Complete 15,613

S6 (lift or lifting or assistive or handling) N2 (device* or instrument* or technolog* or aid or equipment*) Expanders - Apply equivalent subjects
Search modes - Boolean/Phrase Interface - EBSCOhost Research Databases
Search Screen - Advanced Search
Database - CINAHL Complete 15,491

S5 ceiling lift* or floor lifter* or Hoyer lift* Expanders - Apply equivalent subjects
Search modes - Boolean/Phrase Interface - EBSCOhost Research Databases
Search Screen - Advanced Search
Database - CINAHL Complete 80

S4 (MH "Lifting and Transfer Equipment+") Expanders - Apply equivalent subjects
Search modes - Boolean/Phrase Interface - EBSCOhost Research Databases
Search Screen - Advanced Search
Database - CINAHL Complete 1,444

S3 S1 OR S2 Expanders - Apply equivalent subjects
Search modes - Boolean/Phrase Interface - EBSCOhost Research Databases
Search Screen - Advanced Search
Database - CINAHL Complete 14,607

S2 Patient* N3 (Moving* or Lifting* or positioning* or Repositioning* or Handling*) Expanders - Apply equivalent subjects
Search modes - Boolean/Phrase Interface - EBSCOhost Research Databases
Search Screen - Advanced Search
Database - CINAHL Complete 14,208

S1 (MH "Patient Handling+") Expanders - Apply equivalent subjects
Search modes - Boolean/Phrase Interface - EBSCOhost Research Databases
Search Screen - Advanced Search
Database - CINAHL Complete 1,090

Scopus, 145 results
(TITLE-ABS-KEY (patient* W/3 (moving* OR lifting* OR positioning* OR repositioning* OR handling*))) AND ((TITLE-ABS-KEY ("ceiling lift*" OR "floor lifter*" OR "Hoyer lift*") OR TITLE-ABS-KEY (lift OR lifting OR assistive OR handling W/2 device* OR instrument* OR technolog* OR aid OR equipment*))) AND ((TITLE-ABS-KEY (nurse OR nurses) OR TITLE-ABS-KEY (nursing W/1 (staff OR personnel OR assistant*)) OR TITLE-ABS-KEY ("physical therapist*" OR "occupational therapist*" OR physician* OR clinician) OR TITLE-ABS-KEY ((medical OR hospital OR healthcare) W/1 (staff OR personnel OR assistant* OR professional*)))) AND ((TITLE-ABS-KEY (facilitat* OR accept* OR predictor* OR "Use" OR usage) OR TITLE-ABS-KEY (barrier* OR misuse OR "non-use" OR obstacle* OR perception* OR attitude*)))
https://www.scopus.com/results/results.uri?origin=searchhistory&sort=plf-f&src=s&mltEid=&mltAll=t&sid=df3e8fe475877d1f693b0cf2a2ea126b&sot=comb&sdt=comb&sl=772&s=%28TITLE-ABS-KEY%28Patient*+W%2F3+%28Moving*+or+Lifting*+or+positioning*+or+Repositioning*+or+Handling*%29%29%29+AND+%28%28TITLE-ABS-KEY%28%22ceiling+lift*%22+OR+%22floor+lifter*%22+OR+%22Hoyer+lift*%22%29+OR+TITLE-ABS-KEY%28lift+OR+lifting+OR+assistive+OR+handling+W%2F2+device*+OR+instrument*+OR+technolog*+OR+aid+OR+equipment*%29%29%29+AND+%28%28TITLE-ABS-KEY%28nurse+or+nurses%29+OR+TITLE-ABS-KEY%28nursing+W%2F1+%28staff+or+personnel+or+assistant*%29%29+OR+TITLE-ABS-KEY%28%22physical+therapist*%22+or+%22occupational+therapist*%22+or+physician*+or+clinician%29+OR+TITLE-ABS-KEY%28%28medical+or+hospital+or+healthcare%29+W%2F1+%28staff+or+personnel+or+assistant*+or+professional*%29%29%29%29+AND+%28%28TITLE-ABS-KEY%28Facilitat*+or+Accept*+or+Predictor*+or+%22Use%22+or+Usage%29+OR+TITLE-ABS-KEY%28Barrier*+or+Misuse+or+%22non-use%22+or+Obstacle*+or+Perception*+or+Attitude*%29%29%29&txGid=1852f17a755940d498eb7c3ff0cc6de6&sessionSearchId=df3e8fe475877d1f693b0cf2a2ea126b&limit=10

Web of Science Search Strategy (v0.1), # Database: Web of Science Core Collection
- WOS.IC: 1993 to 2023
- WOS.CCR: 1985 to 2023
- WOS.SCI: 1900 to 2023
- WOS.AHCI: 1975 to 2023

- WOS.BHCI: 2005 to 2023
- WOS.BSCI: 2005 to 2023
- WOS.ESCI: 2018 to 2023
- WOS.ISTP: 1991 to 2023
- WOS.SSCI: 1900 to 2023
- WOS.ISSHP: 1991 to 2023

Searches:

1: TS=(Patient* NEAR/3 (Moving* or Lifting* or positioning* or Repositioning* or Handling*)) Results: 11454

2: (TS=(ceiling lift* or floor lifter* or Hoyer lift*)) OR TS=((lift or lifting or assistive or handling) NEAR/2 (device* or instrument* or technolog* or aid or equipment*)) Results: 25624

3: (((TS=(nurse or nurses)) OR TS=(nursing NEAR/1 (staff or personnel or assistant*))) OR TS=(physical therapist* or occupational therapist* or physician* or clinician*)) OR TS=((medical or hospital or healthcare) NEAR/1 (staff or personnel or assistant* or professional*)) Results: 1061247

4: (TS=(Facilitat* or Accept* or Predictor* or "Use" or Usage)) OR TS=(Barrier* or Misuse or "non-use" or Obstacle* or Perception* or Attitude*) Results: 27069730

5: #4 AND #3 AND #2 AND #1 Results: 170
https://www.webofscience.com/wos/woscc/summary/abed8cbd-6f08-4ca2-a34a-e539b1db294b-9caad3fc/relevance/1

Cochrane CENTRAL (AKA trials)

ID	Search	Hits
#1	Patient* NEAR/3 (Moving* or Lifting* or positioning* or Repositioning* or Handling*)	2402
#2	ceiling lift* or floor lifter* or Hoyer lift*	27
#3	(lift or lifting or assistive or handling) NEAR/2 (device* or instrument* or technolog* or aid or equipment*)	1531
#4	#2 OR #3	1554
#5	nurse or nurses	35332
#6	nursing NEAR/1 (staff or personnel or assistant*)	3453
#7	physical therapist* or occupational therapist* or physician* or clinician*	96755
#8	(medical or hospital or healthcare) NEAR/1 (staff or personnel or assistant* or professional*)	9782
#9	#5 OR #6 OR #7 OR #8	129961
#10	Facilitat* or Accept* or Predictor* or "Use" or Usage	647456
#11	Barrier* or Misuse or "non-use" or Obstacle* or Perception* or Attitude*	94077
#12	#10 OR #11	696795
#13	#1 AND #4 AND #9 AND #12 in Trials	6

Trials Register of Promoting Health Interventions (TRoPHI), 1 result

Freetext (All but Authors): "patient lifting" OR "patient handling" OR "patient positioning" "patient repositioning" OR "patient moving"

PAIS Index, 17 results
(Patient* NEAR/3 (Moving* OR Lifting* OR positioning* OR Repositioning* OR Handling*)) AND ((lift OR lifting OR assistive OR handling) NEAR/2 (device* OR instrument* OR technolog* OR aid OR equipment*)) AND (nurse OR nurses OR physical therapist* OR occupational therapist* OR physician* OR clinician*) AND (Facilitat* OR Accept* OR Predictor* OR "Use" OR Usage OR Barrier* OR Misuse OR "non-use" OR Obstacle* OR Perception* OR Attitude*)
https://www.proquest.com/pais/results/1EC1492DFBB4179PQ/1?accountid=15172
